# Detection of frequency-dependent endothelial response to oscillatory shear stress using a microfluidic transcellular monitor

**DOI:** 10.1038/s41598-017-10636-z

**Published:** 2017-08-30

**Authors:** Yoshitaka J. Sei, Song Ih Ahn, Theodore Virtue, Taeyoung Kim, YongTae Kim

**Affiliations:** 1 George W. Woodruff School of Mechanical Engineering, Georgia Institute of Technology, Atlanta, GA 30332 USA; 2 Wallace H. Coulter Department of Biomedical Engineering, Georgia Institute of Technology, Atlanta, GA 30332 USA; 3 Institute for Electronics and Nanotechnology, Georgia Institute of Technology, Atlanta, GA 30332 USA; 4 Parker H. Petit Institute for Bioengineering and Bioscience, Georgia Institute of Technology, Atlanta, GA 30332 USA

## Abstract

The endothelial microenvironment is critical in maintaining the health and function of the intimal layer in vasculature. In the context of cardiovascular disease (CVD), the vascular endothelium is the layer of initiation for the progression of atherosclerosis. While laminar blood flows are known to maintain endothelial homeostasis, disturbed flow conditions including those the endothelium experiences in the carotid artery are responsible for determining the fate of CVD progression. We present a microfluidic device designed to monitor the endothelium on two fronts: the real-time monitoring of the endothelial permeability using integrated electrodes and the end-point characterization of the endothelium through immunostaining. Our key findings demonstrate endothelial monolayer permeability and adhesion protein expression change in response to oscillatory shear stress frequency. These changes were found to be significant at certain frequencies, suggesting that a frequency threshold is needed to elicit an endothelial response. Our device made possible the real-time monitoring of changes in the endothelial monolayer and its end-point inspection through a design previously absent from the literature. This system may serve as a reliable research platform to investigate the mechanisms of various inflammatory complications of endothelial disorders and screen their possible therapeutics in a mechanistic and high-throughput manner.

## Introduction

**Figure 1:**
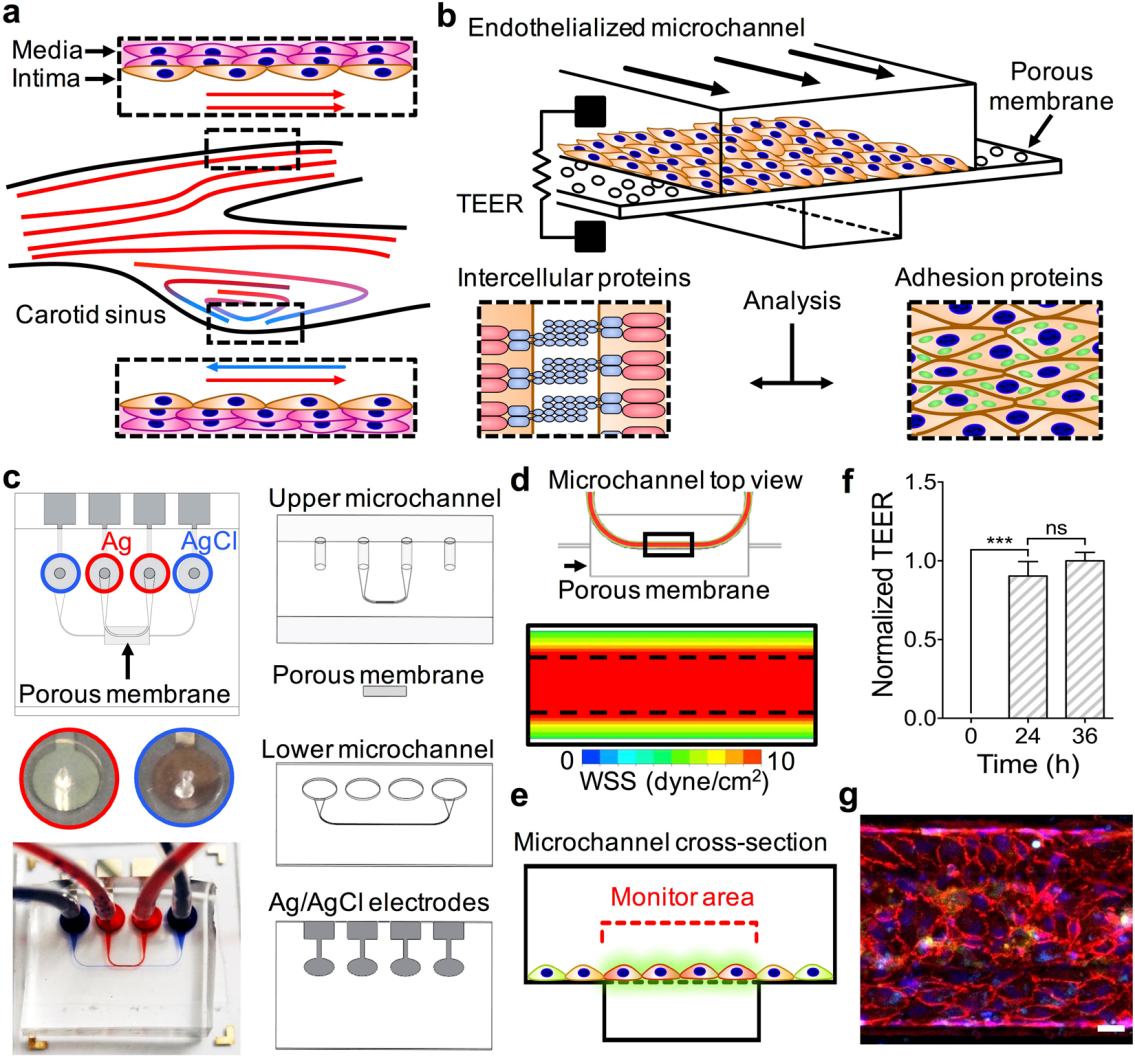
Monitoring of the endothelium for flow-based studies. (**a**) Carotid bifurcation areas of LSS and OSS vary due to expanding sinus geometry. (**b**) A microfluidic transcellular monitor for probing endothelial responses to the flow microenvironment is capable of collecting both monolayer permeability data through TEER and immunostained monolayer images through a transparent porous membrane. (**c**) The device component overview highlighting the Ag/AgCl electrode integration directly into the platform to probe the monolayer growing on the porous membrane. (**d**) CFD simulations in the upper microchannel show the distribution of the wall shear stress (WSS) and the lower microchannel boundary (dotted line). (**e**) Cross-section schematic of upper and lower microchannels shows where the localization of ICAM should occur based on the calculated WSS distribution. (**f**) Monolayer maturity is determined through stabilization of TEER values (N = 3). Plotted as mean ± SEM where * is for p < 0.05. (**g**) Confocal microscopy of an endothelial monolayer stained for adherens junctions (red) and ICAM (green) with DAPI counterstaining on a porous membrane with 8 µm pores. Scale bar is 25 µm.

The vascular endothelium is a vessel-lining barrier that controls the transport of biologically active molecules and regulates inflammation, blood pressure and clotting, and immune cell trafficking. Endothelial homeostasis is critical for maintaining cardiovascular health against the onset of diseases that manifest from cardiovascular disease (CVD)^1^. Atherosclerosis is one such disease that stems from the chronic inflammation of the endothelium^2^. Studies examining the mechanotransduction of endothelial cells have linked endothelial inflammatory responses, including adhesion protein expression and nitric oxide production, to shear stress magnitude and direction^3–5^. Certain areas in the body, like the carotid sinus that has a unique expanding vasculature geometry, have localized disturbed flow acting back and forth against the vascular wall with shear stresses from <10–12 dyne/cm^2^ (1–1.2 Pa) in humans (Fig. 1a)^6, 7^. While endothelial homeostasis is maintained in laminar shear stress (LSS) conditions, the prolonged exposure of the endothelium to oscillatory shear stress (OSS) caused by disturbed flow leads to the chronic inflammatory response, adversely increasing the risk of atherosclerosis and CVD initiation^8–10^.

Despite the understanding of the role of OSS-induced endothelial inflammation in several fatal diseases, challenges remain in sensing the inflamed endothelium under pathological blood flow (i.e. disturbed flow) and delivering sufficient therapeutic agents.

The implementation of atherosclerotic animal models has aided in the study of the disease progression and development of potential therapy options for the disease^11^. One such method to study the atherosclerotic pathology is to use the partial ligation surgery of the left carotid artery (LCA) in apoE−/− mice to apply disturbed flow and the resulting OSS to the endothelium and accelerate atherosclerotic plaque growth^12^. While these findings have improved our understanding of OSS-derived pathology in CVD, further progress in this area is hampered by technical challenges of conducting mechanistic studies on targeted drug discovery and development in the human vasculature. Moreover, extrapolation of animal data to human conditions has been frustratingly controversial, particularly for complex diseases afflicting dynamic microenvironments prevalent with mechanochemical stimuli such as blood flow and inflammatory cytokines^13^. Overcoming traditional *in vitro* models that predominantly rely on static culture of vascular endothelial cells, current advanced *in vitro* studies on endothelial mechanotransduction have taken the magnitude and direction of OSS into consideration to complement existing animal model research^3, 4, 8, 9, 14–16^. The frequencies typically associated to be characteristic of disease-causing OSS are based on measured physiological values *in vivo*; this value is about 5 Hz in mice, whereas in humans this level drops to approximately 1 Hz^3, 10, 17^. Despite sophisticated approaches to apply OSS *in vitro*, variations in endothelial cell responses to slight changes in the disturbed flow pattern may not be adequately reflected for studying certain mechanisms such as the dynamic interaction of the vascular endothelium with drugs *in vivo* ^18, 19^. More importantly, little work has questioned whether the endothelial response is affected by the frequency of OSS, which may be key to influence expression of pro-inflammatory proteins like VCAM-1, ICAM-1, or IL-8^20, 21^. Consequently, emerging technologies like biomimetic microfluidic platforms are being developed to address questions like these by implementing physiological and pathological conditions in a minimalist *in vitro* setting of OSS-induced vascular complications.

Microfluidic platforms, some of which are called organ-on-a-chip systems, provide an unprecedented ability to control the mechanical and chemical microenvironment in a spatial and temporal fashion, enabling the studies of dynamic cellular responses to a variety of mechanical and chemical stimuli^22^. The capabilities to measure cellular responses from dynamic environments typically found in physiological systems, as well as the potential for high-throughput applications, make microfluidic technology a suitable medium through which to study shear-dependent endothelial responses with spatiotemporal manners and high precision. Microfluidic platforms have also grown to include screening tools to accelerate the development of therapeutics in physiological disease settings^23^. We report on the dependency of the endothelial inflammatory response to changes in OSS frequencies as presented through the implementation of a microfluidic transcellular monitor to dynamically examine the permeability and adhesion molecule expression level of an endothelial monolayer. Additionally, we report and address the challenges of widely used existing double-layered endothelialized microfluidic platforms; the endothelium near the channel wall fails to replicate physiologically relevant endothelial morphology and function in response to the application of shear, compromising the accuracy to investigate both the effect of mechanical and chemical cues on the endothelium and the translocation of nanoparticles across the endothelial barrier^24^. By imposing the accurate shear stress on primary human aortic endothelial cells (HAECs), as well as monitoring the endothelial permeability, our microfluidic transcellular monitor may provide a simple yet reliable platform to: (i) examine the underlying mechanisms by which vascular endothelial disorders occur and lead to complicated inflammatory pathologies, and (ii) efficiently test the efficacy of potential drug candidates targeting the dysfunctional endothelium in various mechanically (shear stress) and chemically (pro-inflammatory cytokines) stimulated pathophysiologically relevant conditions.

## Results

### Microfluidic transcellular monitor design

The microfluidic transcellular monitor platform was designed to study the endothelial responses through electrical measurements and microscopy data that track the intercellular permeability and overall visual health of the monolayer in a multi-layer design (Fig. 1b). High-throughput and real-time monitoring of the endothelium was accomplished through an array of devices implementing the transendothelial electrical resistance (TEER) across the upper and lower microchannels using Ag/AgCl electrodes integrated into the device (Fig. 1c and Supplementary Fig. S1). This double-layered microfluidic platform uses a high endothelial cell seeding density to allow for the uniform coverage of endothelial cells across the microchannel. To address the issue of the non-homogenous morphological and functional responses of endothelial cells due to their proximity to the sharp corner of the microchannel wall and better model the vascular endothelium, we performed computational fluid dynamics (CFD) analyses to find that the endothelial monolayer would only experience the greatest directional changes in response to flow within the middle 50% of the upper microchannel width (Fig. 1d). We validated these CFD simulation results using human umbilical vein endothelial cells (HUVECs), a widely used endothelial cell type *in vitro*, cultured in the upper microchannel geometry on a glass substrate to show nearly a 30% decrease in mean cell area for cells growing in the middle of the microchannel under flow, the absence of which resulted in an insignificant difference in the mean cell area between the cells growing in the two regions (Supplementary Fig. 2). Taking into account both the TEER dependency on the overlapping area of the upper and lower microchannels and the CFD simulation results, the upper microchannel was designed to have twice the width of the lower microchannel (200 µm and 100 µm, respectively) so that we could monitor an area that would only include the middle 50% region of the upper microchannel (Fig. 1e). With this microchannel configuration, we were able to efficiently inspect the HAEC monolayer growth and maturation using TEER, the leveling-off of which identified the monolayer maturity with high reproducibility (Fig. 1f). Visual inspection of the HAEC monolayer through confocal microscopy was used in conjunction with TEER to gain a more representative understanding over how the endothelial monolayer responded to our flow-based stimuli (Fig. 1g).

### Tailored platform more suitable for endothelial response monitoring

**Figure 2:**
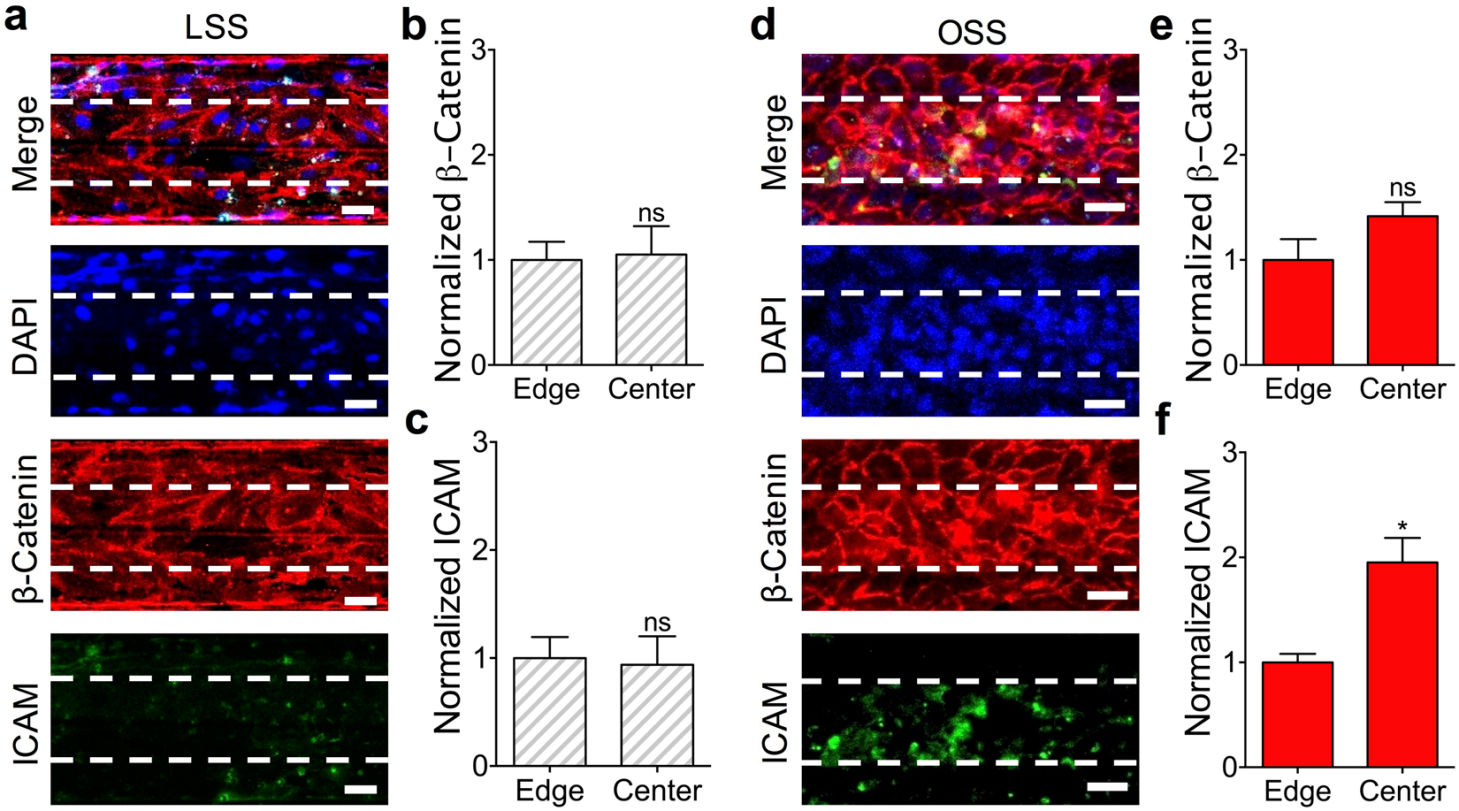
Microfluidic transcellular monitor design. (**a**) Confocal microscopy images were taken through the underside of the porous membrane of an HAEC monolayer cultured in the upper microchannel under LSS at 10 dyne/cm^2^ that was stained for β-catenin and ICAM (lower microchannel outlined with dotted white lines). Scale bar is 40 µm. (**b**) β-catenin channel intensity measurements and (**c**) ICAM channel intensity measurements for the LSS case comparing the center and edge regions normalized to the edge region (N = 3). (**d**) Confocal microscopy images were taken through the underside of the porous membrane of an HAEC monolayer cultured in the upper microchannel under OSS at 1 Hz that was stained for β-catenin and ICAM (lower microchannel outlined with dotted white lines). Scale bar is 40 µm. (**e**) β-catenin channel intensity measurements and (**f**) ICAM channel intensity measurements for the OSS case comparing the center and edge regions normalized to the edge region (N = 3). Plotted as mean ± SEM where * is for p < 0.05.

Using this new design that can accurately impose both the specific quantity and direction of shear stress on the endothelium cultured in the middle microchannel, we then investigated whether our microfluidic transcellular monitor was capable of accurately reconstituting pathophysiological conditions caused by shear stress profiles. We first cultured HAECs to form a monolayer to be exposed to LSS of +10 dyne/cm^2^ in order to investigate whether or not there is a locally distinct expression of EC proteins like the adhesion protein ICAM and the adherens junction protein β-catenin in the center region (defined as the middle 50% region) of the upper microchannel (Fig. 2a). Comparing the center region to the remaining area of the upper microchannel (defined as the edge region) yielded a non-significant difference in the β-catenin (Fig. 2b) and ICAM intensity (Fig. 2c). We then examined this effect with an HAEC monolayer exposed to OSS of +10 and −9 dyne/cm^2^ at 1 Hz (Fig. 2d). The comparison of the center region to the edge region yielded a non-significant difference in β-catenin intensity (Fig. 2e). However, there was nearly double the ICAM intensity expressed in the center region compared to that in the edge region (Fig. 2f). Following the verification of our simulation results with the observation of ICAM localization to the center region of the microchannel, the confocal imaging area for comparisons across devices with varying shear stress conditions was then defined to only include the center region due to the verified non-negligible effects of the microchannel wall. In following the same design intent, the choice to make the lower microchannel half as wide as the upper microchannel was to minimize the effect of the wall on TEER measurements. This is especially important for the accurate monitoring of the endothelial permeability in response to varying shear stress conditions, such as shear oscillation frequency, due to the direct influence the overlapping area of the upper and lower microchannels has on TEER measurements^25^.

### Endothelial response is dependent on the OSS frequency

**Figure 3:**
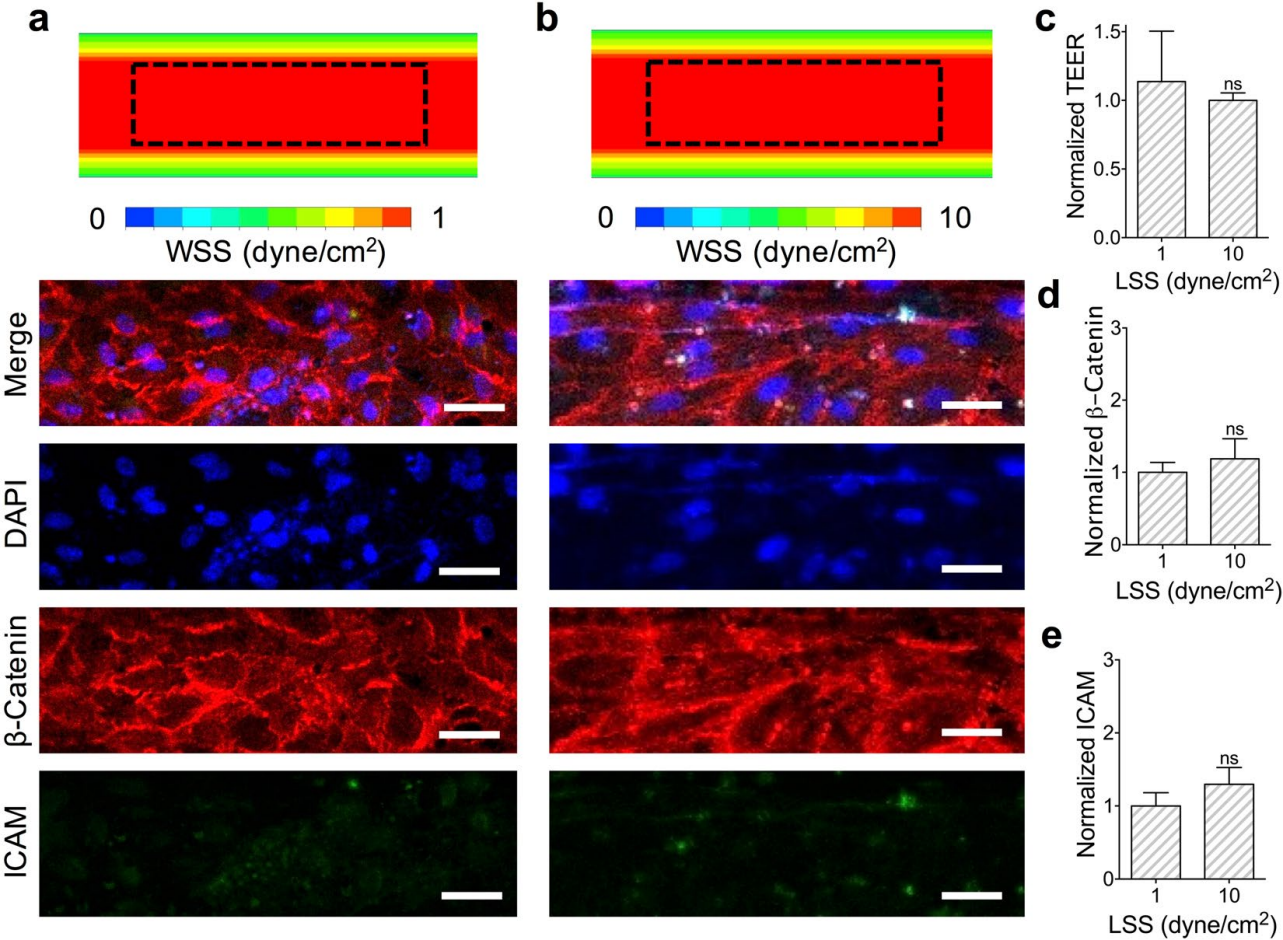
Comparing LSS cases for +1 and +10 dyne/cm^2^. (**a**) Upper microchannel WSS distribution and confocal microscopy images of HAEC monolayers cultured under +1 dyne/cm^2^ and (**b**) +10 dyne/cm^2^ as viewed through the underside of the porous membrane. Confocal images used for comparison were taken from a region in the center of the upper microchannel (outlined). Scale bar is 40 µm. (**c**) Normalized TEER of HAEC monolayer cultured under +1 or +10 dyne/cm^2^. (**d**) β-catenin channel intensity measurements and (**e**) ICAM channel intensity measurements for the LSS cases comparing +1 and +10 dyne/cm^2^ normalized to 1 dyne/cm^2^ case (N = 3). Plotted as mean ± SEM where * is for p < 0.05.

**Figure 4:**
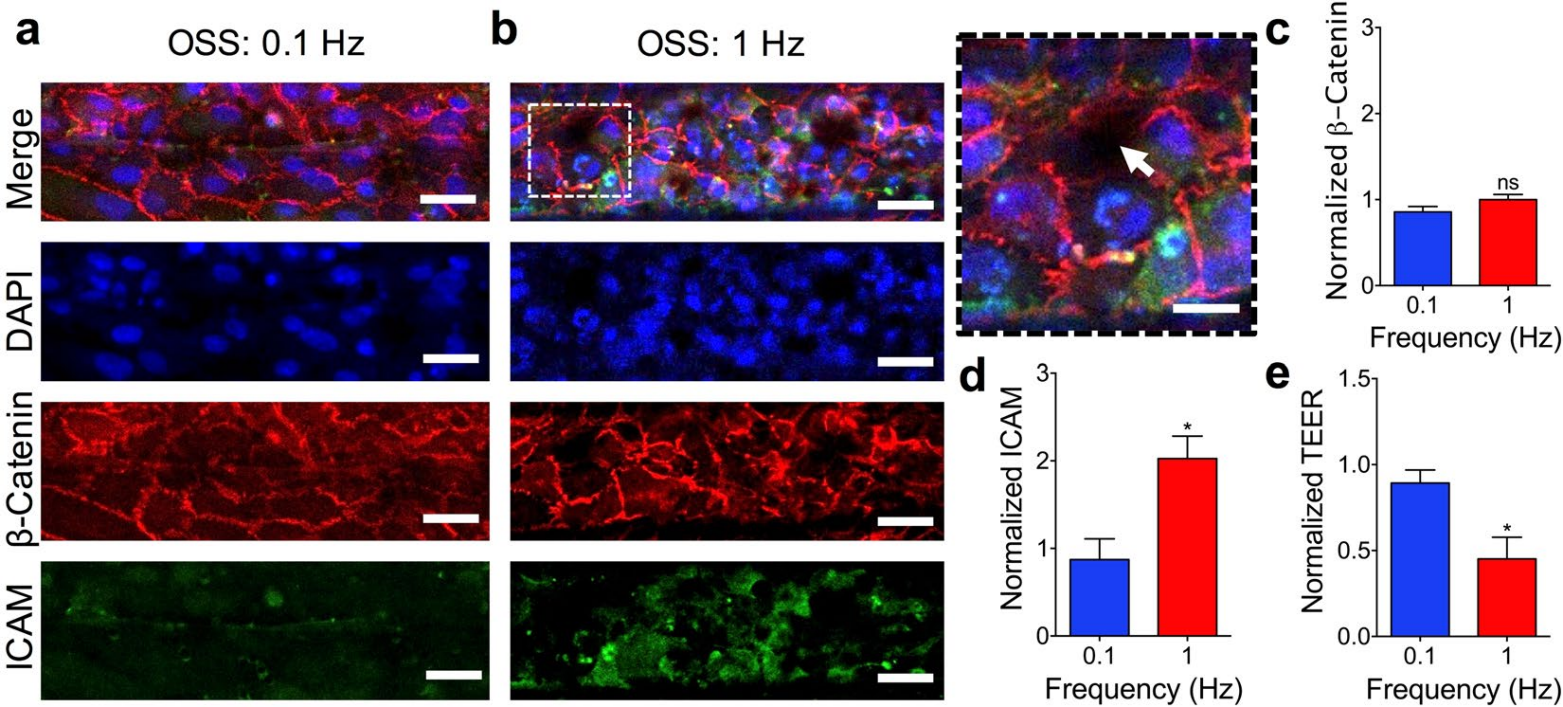
Monitoring of endothelial frequency response. (**a**) Top view confocal images of HAEC monolayer in the center region cultured under 0.1 Hz. (**b**) Top view confocal images of HAEC monolayer in the center region cultured under 1 Hz with disrupted endothelial junction (white arrow). Scale bar is 40 µm and 20 µm for enlarged image. (**c**) β-catenin intensity channel measurements for the OSS cases comparing 0.1 and 1 Hz normalized to 10 dyne/cm^2^ case (N = 3). (**d**) ICAM intensity channel measurements for the OSS cases comparing 0.1 and 1 Hz normalized to 10 dyne/cm^2^ LSS case (N = 3). (**e**) Normalized TEER of HAEC monolayer after 36 hours of culture under 12 hours of 0.1 or 1 Hz normalized to the 10 dyne/cm^2^ LSS case. Plotted as mean ± SEM where * is for p < 0.05.

The carotid artery is a region of interest in CVD research due to its unique geometry to produce locally recirculating or disturbed flows. The shear stresses in these regions average to about 10 dyne/cm^2^, which is a commonly used shear stress value with HAECs under laminar flow *in vitro* ^6, 7, 26, 27^. Shear stresses of +10 and +10/−9 dyne/cm^2^ were used for the LSS and OSS conditions respectively to minimize variations in the flow conditions to be in OSS frequency while still allowing the refreshment of media in the devices. The net shear stress magnitude to be used between the OSS and LSS cases, +1 and +10 dyne/cm^2^ respectively, were compared through CFD-calculated WSS distributions, β-catenin intensity measurements, and ICAM intensity measurements in the center channel region (Fig. 3a and b). The HAEC monolayers matured in 24 hours under LSS of +10 dyne/cm^2^ (Fig. 1f), after which the various shear conditions were imposed for 12 hours (Supplementary Fig. S3). Between the +1 and +10 dyne/cm^2^ cases under LSS, the differences in TEER, β-catenin intensities, and ICAM intensities were insignificant (Fig. 3c–e). For the examination of the OSS frequency effects, the HAEC monolayers were cultured under 0X (LSS), 0.1X (OSS), and 1X (OSS) of the 1 Hz OSS frequency typically used *in vitro* to approximate the average resting heart rate of an individual^3^. The immunostaining comparisons showed disruptions in the endothelial monolayer and increased ICAM expression for 1 Hz case (Fig. 4a and b). Measuring the ICAM and β-catenin channel intensities revealed that while there was no significant difference in β-catenin expression, the 1 Hz case had a significantly higher expression of ICAM (Fig. 4c and d). Monitoring of the endothelial permeability revealed that the TEER significantly decreased only for the HAEC monolayers exposed to OSS of 1 Hz compared to HAEC monolayers exposed to OSS of 0.1 Hz (Fig. 4e). TEER measurements with iMAECs instead of HAECs also showed the TEER to drop significantly after passing a certain frequency threshold (Supplementary Fig. S4).

## Discussion

The design of the microfluidic transcellular monitor was based on the rationale that the endothelial cells cultured closer to the side walls of the microchannel with a rectangular cross section would exhibit different morphologies from experiencing different shear stress profiles; in our case specifically, the oscillatory fluid flow would have a more observable effect on endothelial cells cultured in the middle of the microchannel. Our results change the status quo of key design parameters to keep in mind when concerning microfluidic analyses of endothelial monolayers; there is now a cause for future double-layer microchannel platforms to more carefully take the microchannel configuration into consideration to minimize the influence of the microchannel wall on the experiment. With TEER measurements depending on the overlapping microchannel area, we took this design criteria into consideration by limiting the lower microchannel width to be half that of the upper microchannel^25^. The cultures of HUVECs under LSS and the cultures of HAECs under LSS and 1 Hz OSS were immunostained to validate our rationale and simulations with comparisons of mean cell area, β-catenin expression, and ICAM expression between the center (middle 50%) and edge regions (remaining 25% area on either side) of the upper microchannel.

Our validations of the insignificant differences between the net shears of +1 and +10 dyne/cm^2^ determined that the differences observed between our cases were due to the different flow frequency cases and not in the shear magnitudes. The monitoring of the endothelium during different OSS frequencies showed consistent results across both human and mouse endothelial cells, indicating that the endothelial sensitivity to OSS frequency was not species-limited. Our results also indicate that there is a frequency threshold that must be reached in order to stimulate endothelial inflammation with OSS, suggesting that there may be a range of frequencies over which endothelial cells will respond in a pro-inflammatory manner. While it is still unclear as to what the breadth of this endothelial pro-inflammatory frequency range includes, comparisons of our results with prior studies on the risks of resting heart rate and CVD suggest that higher resting heart rates, and thus potentially higher OSS frequencies, have higher risks of experiencing a CVD-related episode^28, 29^.

Our findings suggest that the inflammatory response of endothelial cells to OSS may be triggered by meeting a minimum frequency requirement. We demonstrated the design rationale behind our platform to take advantage of its capabilities to detect changes in both monolayer permeability and inflammatory marker expression in response to changes in OSS frequency. Future work to consider with this device would be to explore the extent of the frequency dependency by pairing the device with a more sophisticated fluid flow controller to impose high frequency flows, as well as to screen extensive libraries of nanomedicines and therapeutics targeting the endothelium to rapidly identify and catalogue endothelial responses^30^. The translation of these results across humans and mice may also be further pursued to help bridge the gap between human and animal models.

## Methods

### Materials and chemicals

The reagent (3-aminopropyl)triethoxysilane (APTES) was purchased from Sigma-Aldrich Co. LLC. (St. Louis, MO). Polydimethylsiloxane (PDMS) was purchased as Sylgard 184 from Dow Corning (Midland, MI). The photoresist SU-8 and its developer were purchased from MicroChem Corp (Westborough, MA). HAECs and HUVECs were purchased from ATCC (Manassas, VA) and ScienCell Research Laboratories (Carlsbad, CA) respectively. The iMAECs were generously donated from the lab of Dr. Hanjoong Jo.

### Microfluidic transcellular monitor design and fabrication

The microfluidic transcellular monitor was first conceptualized using the CAD software SolidWorks from Dassault Systemes SolidWorks Corp. (Paris, France), after which we conducted computational flow studies using the Fluent solver (ANSYS, Inc. Concord, MA). The dimensions of the upper and lower microchannels were 200 µm × 100 µm and 100 µm × 50 µm respectively (width × height). All simulations were solved for a single fluid with the Newtonian properties of water and using a structured mesh for the device microchannel^31, 32^. The boundary condition at the device inlet was set to 20 µL/min and 2 µL/min for the average +10 and +1 dyne/cm^2^ conditions respectively to examine the distribution of the WSS in the cell-culture region of the upper microchannel. Fabrication of the device started with Ag electrodes patterned onto glass slides using metal evaporation deposition^33^. The thin PDMS sheet for the lower microchannel was made by spin coating PDMS to 250 µm in height onto an SU-8 patterned silicon wafer. The upper microchannel was constructed using standard soft lithography to a patterned wafer. An 8 µm pore polycarbonate membrane (Sterlitech Corp, Kent, WA) was then treated in a 5% APTES solution at 80 °C for device assembly prior to device housing^34^. The lower microchannel AgCl electrodes were produced using household bleach to soak the Ag electrodes for 1 hour at room temperature^35^. The fabricated device was housed in a polystyrene box for microfluidic cell culture using syringe pumps (Supplementary Fig. S1).

### Microfluidic endothelial cell culture preparation

Prior to seeding cells into devices, HAECs, iMAECs, and HUVECs were cultured in EGM-2 (Lonza, Basel, Switzerland) and complete endothelial growth medium (DMEM with 10% FBS, 1% endothelial cell growth supplement (ECGS), and 1% penicillin and streptomycin) respectively. Cell media was replaced every 3 days, with cultures being split at confluence. The device, housing, and tubing were sterilized with 70% ethanol and washed with DI-water and PBS (1X, pH 7.4) prior to filling the microchannels with a fibronectin solution (50 µg/mL in PBS) and incubating the devices for 2 hs at 37 °C. After the fibronectin incubation period, the channels were rinsed with PBS prior to seeding cells into the devices at a concentration of approximately 1 × 10^7^ cells/mL. Following a 1 h incubation at 37 °C, devices were connected to Harvard Apparatus (Holliston, MA) PhD Ultra syringe pumps and exposed to 20 µL/min (HAECs and HUVECs) or 14 µL/min (iMAECs) while incubating at 37 °C. To study the effects of OSS frequency, monolayers were cultured for 24 hours and 48 hours for HAECs and iMAECs respectively before applying the different shear frequencies (Supplementary Figs S3 and S4).

### TEER measurements

TEER measurements were taken every 12–24 hours at 37 °C using the EVOM2 volt-ohmmeter (World Precision Instruments, Sarasota, FL). A potential source of error is from noise in the TEER measurements, which was reduced by averaging multiple readings for each device at every time point, and by taking all measurements at the same temperature inside of a cell culture incubator, as TEER can also be affected by temperature fluctuations. Measurements were normalized to the mature monolayer value in each device relative to when there were no cells in the device (0 h).

### Immunostaining of endothelial cells

Cell fixation was done using 4% paraformaldehyde (PFA) in PBS before permeabilizing in 0.1% triton. Samples were blocked with 1% bovine serum albumin (BSA) in PBS. Antibody incubation was with anti-β-catenin antibodies (ab32572, Abcam, Cambridge, UK) and Alexa Fluor 488 conjugated anti-ICAM-1 antibodies (322713, BioLegend, San Diego, CA) at 4 °C overnight followed by Alexa Fluor 647 conjugated secondary antibodies (ab150075, Abcam). Confocal microscopy imaging of the stained devices was done with a Zeiss LSM 700 microscope (Zeiss, Oberkochen, Germany).

### Image analysis

Confocal images of each device were analyzed using the regions of interest (ROI) manager in ImageJ. For comparisons of the center and edge regions of the microchannel, the microchannel was parsed into 8 sections; the center 4 sections were used to define the center ROI, and the outer 4 sections were used to define the edge ROI. For the frequency dependency analyses, only the center ROI of the microchannels was compared. The individual channels for ICAM and β-catenin were used for ROI comparisons. HUVEC areas were calculated using ImageJ ROI manager, where cells were sorted into center and edge regions based on the area the cell occupied the most.

### Statistical analysis

Statistical analyses were run in Prism 6 (GraphPad Software Inc, La Jolla, CA) and significance was defined as p < 0.05 (*). ANOVA tests were used to compare the TEER and immunostaining results of 0X, 0.1X, and 1X frequency cases, and t-tests were used for the +1/+10 dyne/cm^2^ LSS comparisons and the center/edge comparisons.

## Electronic supplementary material

Supplementary Information
