# Supplementary material for: Detection of frequency-dependent endothelial response to oscillatory shear stress using a microfluidic transcellular monitor
Source: Sci Rep. 2017 Aug 30;7:10019. doi: 10.1038/s41598-017-10636-z (PMC5577378; doi:10.1038/s41598-017-10636-z)
Supplement: Supplementary file 1 — Supplementary Information [file 41598_2017_10636_MOESM1_ESM.pdf]

Supplementary Information

Detection of frequency-dependent endothelial response to oscillatory shear stress using a microfluidic transcellular monitor

Yoshitaka J Sei[ad], Song Ih Ahn[ad], Theodore Virtue[a], Taeyoung Kim[a], and YongTae Kim[abcd*]

[a]George W. Woodruff School of Mechanical Engineering, [b]Wallace H. Coulter Department of Biomedical Engineering, [c]Institute for Electronics and Nanotechnology, [d]Parker H. Petit Institute for Bioengineering and Bioscience, Georgia Institute of Technology, Atlanta, GA 30332, USA

*Corresponding author: ytkim@gatech.edu

George W. Woodruff School of Mechanical Engineering, Wallace H. Coulter Department of Biomedical Engineering, Institute for Electronics and Nanotechnology (IEN), Parker H. Petit Institute for Bioengineering and Bioscience (IBB), Georgia Institute of Technology, 345 Ferst Drive (Rm 3134), Atlanta, GA 30332, Office: 404.385.1478, Fax: 404.385.8535

Supplementary Figure count: 4

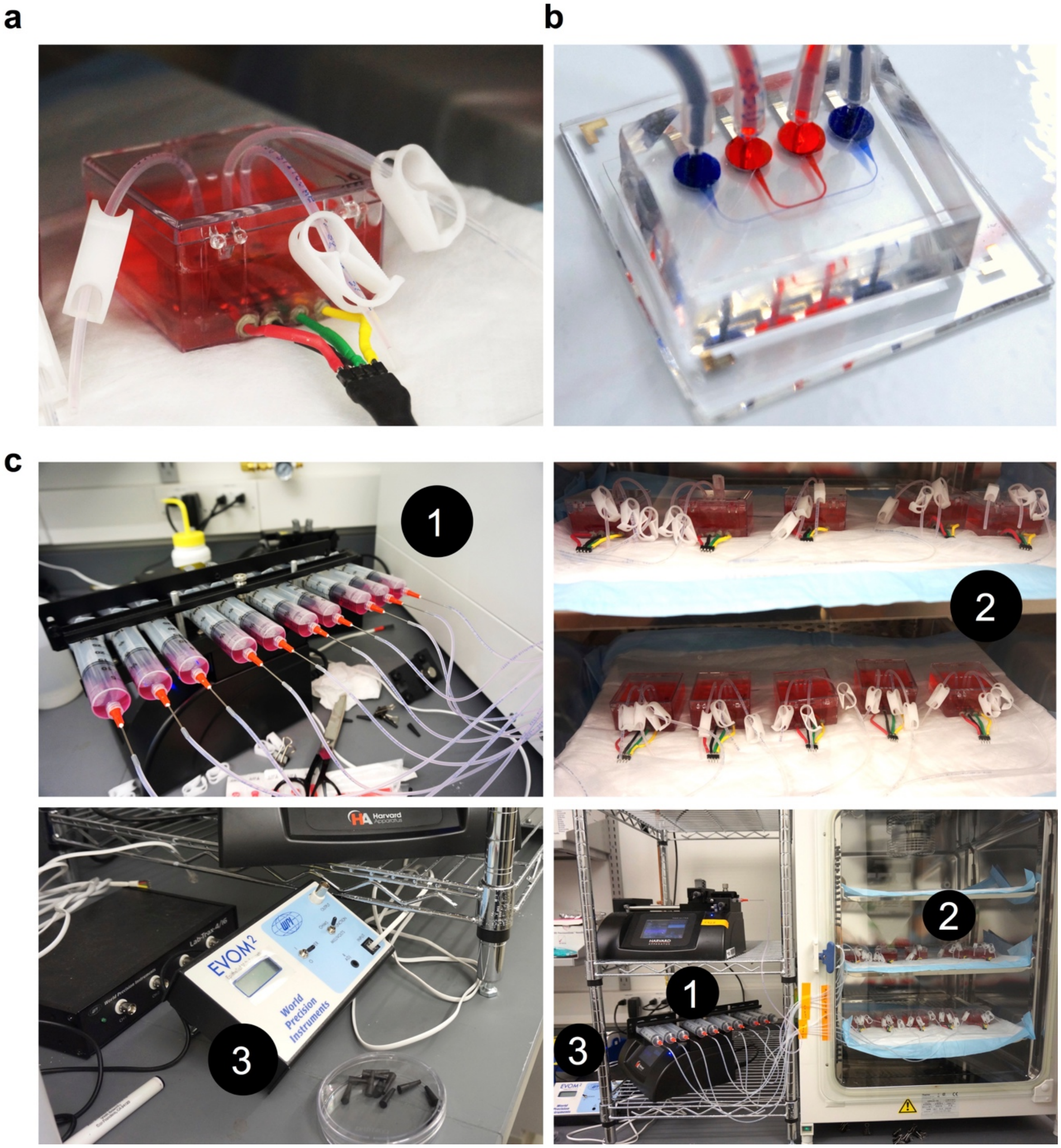

Figure S1. Images of microfluidic transcellular monitor. (**a**) The device during cell culture is enclosed in a polystyrene box with a wire connector interface to communicate with an EVOM2 voltohmmeter. (**b**) Image of device highlighting the upper microchannel (red) and lower microchannel (blue) which are separated by a transparent porous membrane. (**c**) High throughput experimental setup using (1) multi-rack syringe pumps to apply flow to (2) incubated devices that are monitored with the (3) EVOM2 voltohmmeter.

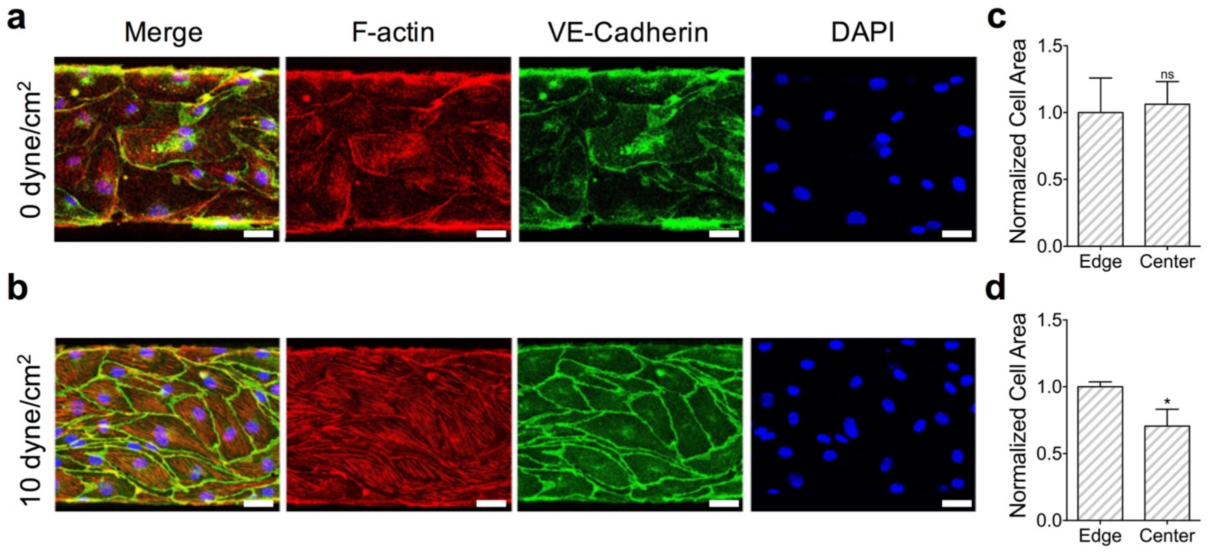

Figure S2. The effect of the microchannel wall. (**a**) Confocal images of HUVECs that were cultured in a single layer device with the upper microchannel geometry and a glass bottom substrate under 0 dyne/cm^2 and (**b**) +10 dyne/cm^2 LSS conditions. Scale bar is 25 μm. (**c**) The mean area was compared between cells growing near the edge of the channel and cells growing in the center of the channel for 0 dyne/cm^2 and (**d**) +10 dyne/cm^2 normalized to the mean cell area at the edge of the channel (N = 7-8). Plotted as mean ± SEM where * is for $p < 0.05$.

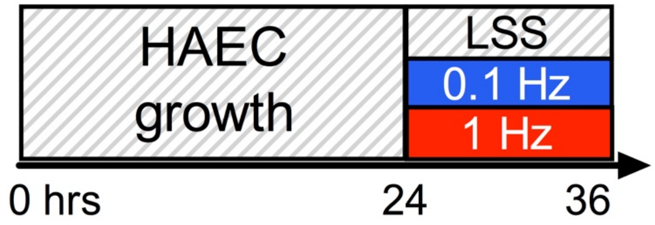

Figure S3. OSS culture timeline for HAECs. (**a**) Timeline of monolayer growth in devices for HAECs to compare OSS frequency effects on a mature endothelial monolayer.

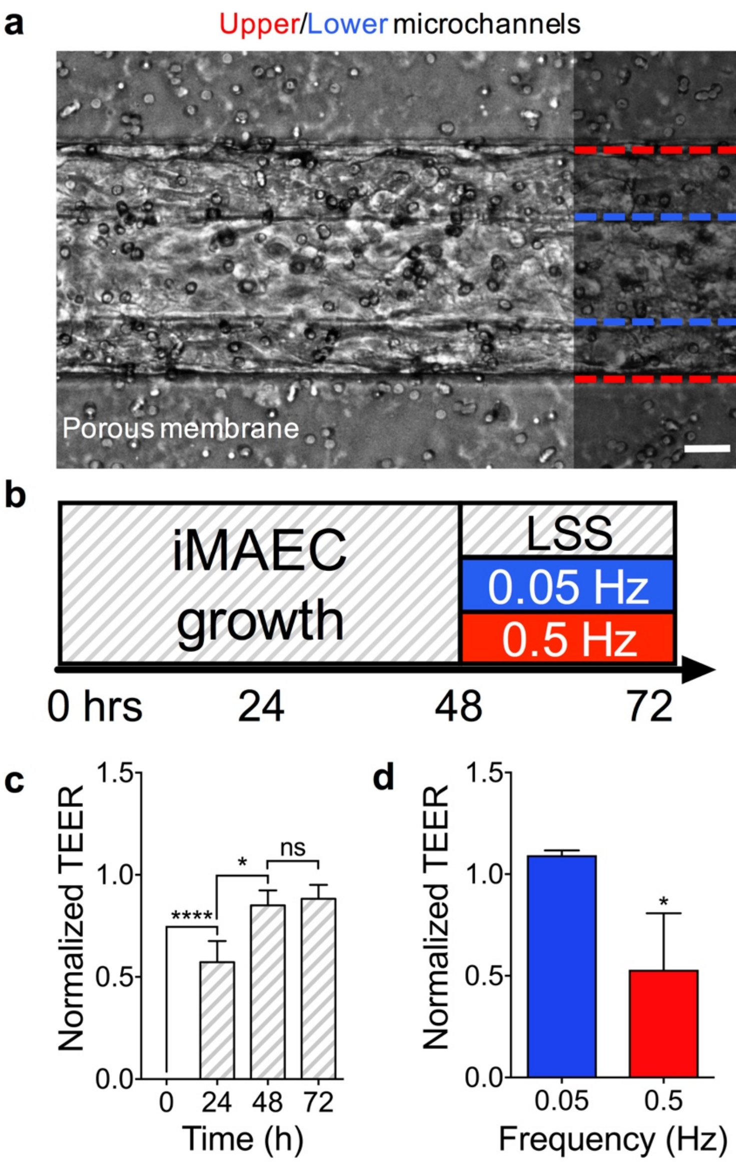

Figure S4. OSS frequency dependent response in iMAECs. (a) Contrast image of an iMAEC monolayer growing in a device with the upper (red) and lower (blue) microchannels highlighted. Scale bar is 40 µm. **(b)** Timeline of monolayer growth in devices for iMAECs to compare OSS frequency effects on a mature endothelial monolayer. **(c)** Normalized TEER tracking of iMAEC monolayer growth over 72 hours (N = 4-13). **(d)** Normalized TEER of iMAEC monolayer after 72 hours of culture under 24 hours of 0.05 or 0.5 Hz normalized to the LSS case (N = 3-5). Plotted as mean ± SEM where * is for $p < 0.05$.
